# Peri-pubertal exposure to testicular hormones organizes response to novel environments and social behaviour in adult male rats

**DOI:** 10.1016/j.yhbeh.2015.07.003

**Published:** 2015-07

**Authors:** Gillian R. Brown, Kyle D. Kulbarsh, Karen A. Spencer, Camille Duval

**Affiliations:** School of Psychology & Neuroscience, University of St Andrews, UK

**Keywords:** Adolescence, Testosterone, Sex differences, Exploration, Sexual behaviour, Castration

## Abstract

Previous research has shown that exposure to testicular hormones during the peri-pubertal period of life has long-term, organizational effects on adult sexual behaviour and underlying neural mechanisms in laboratory rodents. However, the organizational effects of peri-pubertal testicular hormones on other aspects of behaviour and brain function are less well understood. Here, we investigated the effects of manipulating peri-pubertal testicular hormone exposure on later behavioural responses to novel environments and on hormone receptors in various brain regions that are involved in response to novelty. Male rodents generally spend less time in the exposed areas of novel environments than females, and this sex difference emerges during the peri-pubertal period. Male Lister-hooded rats (*Rattus norvegicus*) were castrated either before puberty or after puberty, then tested in three novel environments (elevated plus-maze, light–dark box, open field) and in an object/social novelty task in adulthood. Androgen receptor (AR), oestrogen receptor (ER1) and corticotropin-releasing factor receptor (CRF-R2) mRNA expression were quantified in the hypothalamus, hippocampus and medial amygdala. The results showed that pre-pubertally castrated males spent more time in the exposed areas of the elevated-plus maze and light–dark box than post-pubertally castrated males, and also confirmed that peri-pubertal hormone exposure influences later response to an opposite-sex conspecific. Hormone receptor gene expression levels did not differ between pre-pubertally and post-pubertally castrated males in any of the brain regions examined. This study therefore demonstrates that testicular hormone exposure during the peri-pubertal period masculinizes later response to novel environments, although the neural mechanisms remain to be fully elucidated.

## Highlights

• Pre- and post-pubertally castrated male rats were tested in novel environments and with novel opposite-sex conspecifics.
• Males that were castrated before puberty spent more time in the exposed areas of novel environments.
• Males that were castrated after puberty spent more time investigating opposite-sex conspecifics.
• No differences were found in hormone receptor gene expression levels in the brain between treatment groups.
• Exposure to testicular hormones during the peri-pubertal period organizes later behaviour.

## Introduction

Puberty is characterised by increased circulating levels of gonadal hormones, such as testosterone and estradiol, and is accompanied by a suite of physical and behavioural changes in both human beings and non-human animals (Blakemore, 2008; Spear, 2000). The brain also undergoes substantial reorganization during this period of life in a range of species (Andersen, 2003; Brown and Spencer, 2013; Goddings et al., 2014). Recent studies of laboratory rodents have shown that gonadal hormones can direct brain development during the peri-pubertal period by influencing neurodevelopmental processes, such as cell proliferation and axon myelination, with long-term implications for brain structure and function (e.g., Ahmed et al., 2008; De Lorme et al., 2012a; Yates and Juraska, 2008). In addition, experimental studies on rodents have shown that exposure to gonadal hormones during the peri-pubertal period has long-term effects on behaviour; for example, male hamsters that were castrated before puberty had lower sexual responsiveness to female conspecifics, and showed less aggression to same-sex conspecifics, in adulthood than males that were castrated after puberty (e.g., Schulz et al., 2004; Schulz and Sisk, 2006). Therefore, the developing brain, and its behavioural outputs, can be described as being sensitive to the long-term, ‘organizational’ effects of gonadal hormones during the peri-pubertal period (Schulz et al., 2009; Sisk and Zehr, 2005; Juraska et al., 2013).

While most of the recent studies on the organizational effects of peri-pubertal hormone exposure have focused on sexual and social behaviour, early studies provided preliminary evidence that removal of testicular hormones during the peri-pubertal period also leads to a more female-typical response to novel environments in adulthood (Brand and Slob, 1988; Primus and Kellogg, 1989, 1990; Swanson, 1966). Female rodents commonly ambulate more than males in novel, ‘open field’ environments (e.g., Slob et al., 1981; Hyde and Jerussi, 1983), and these early studies reported that adult male rats that had been castrated before puberty ambulated more in open field environments, and exhibited more social interactions in novel environments, than males castrated after puberty (Brand and Slob, 1988; Primus and Kellogg, 1989, 1990). In general, females exhibit higher locomotor responses than males to a range of novel environments and spend more time than males in the exposed areas of novel arenas such as elevated plus-mazes and light–dark boxes (e.g., Johnston and File, 1991; Ramos et al., 2002; reviewed by Joel and Yankelevitch-Yahav, 2014). These behavioural sex differences in response to novel environments emerge during the peri-pubertal period (e.g., Imhof et al., 1993; Lynn and Brown, 2009, 2010) and have been shown to be sensitive to the presence of testosterone during the first few days of postnatal life (e.g., Lucion et al., 1996). However, whether peri-pubertal testosterone exposure has organisational effects on response to novel environments remains unclear, as other studies have reported no effect of pre-pubertal castration on later open field behaviour (e.g., Bengelloun et al., 1976; Stewart and Cygan, 1980), and the effects of peri-pubertal gonadal hormone exposure on adult behaviour in other novel environments have not yet been investigated.

The aim of this study was to examine the organizational effects of exposure to testicular hormones during the peri-pubertal period on later response to novel environments in male rats using a number of behavioural tasks that evoke sex differences in response, namely an open field (OF) environment, an elevated plus-maze (EPM) and a light–dark (LD) box (Joel and Yankelevitch-Yahav, 2014; Simpson and Kelly, 2012). Male Lister-hooded rats that had been castrated either before or after puberty were tested on these behavioural tasks in adulthood. Males that had not been exposed to testosterone during the peri-pubertal period (i.e., castrated before puberty) were predicted to locomote more in the apparatus, and spend more time in the exposed areas (centre of OF, open arms of EPM, light area of LD box), than males that had experienced normal levels of circulating testosterone during the peri-pubertal period (i.e., castrated after puberty). These predictions were based on the well-documented behavioural sex differences in these tasks and on previous research on the organizational effects of testosterone during earlier stages of life, which has shown that exposure to testosterone during the first few days of life masculinizes locomotor activity and time in exposed areas of novel environments in adulthood (e.g., Broida and Svare, 1984; Lucion et al., 1996; Zuloaga et al., 2011). In addition, the effects of pre- and post-pubertal castration on response to novel objects and opposite-sex partners were examined, given that suppression of pubertal testosterone reduces preference for novelty in male rats when tested during the peri-pubertal period (Cyrenne and Brown, 2011).

The long-term impacts of pre- and post-pubertal castration on brain development were investigated by measuring steroid hormone receptor levels in several brain regions that are known to be involved in behavioural and neuroendocrine responses to novelty, specifically the ventromedial hypothalamus, hippocampus and medial amygdala (Kabbaj and Akil, 2001; Shin and Liberzon, 2010; Singewald, 2007). Gonadal hormones can influence behaviour and brain development via modulation of steroid hormone receptors levels (Juraska et al., 2013), and recent studies have confirmed that testosterone exposure during the peri-pubertal period can alter steroid receptor levels in adulthood (e.g., Nuruddin et al., 2013; Purves-Tyson et al., 2012; Romeo et al., 2000), potentially leading to life-long changes in steroid hormone responsiveness. For example, androgen receptor levels in the hypothalamus were found to be higher in adult male hamsters that had been castrated before puberty than in males that had been castrated post-pubertally (Romeo et al., 2000). In adult rodents, sex differences in both androgen and oestrogen receptor levels have been reported in the hypothalamus, hippocampus and medial amgydala (Simerly et al., 1990), and these brain regions undergo sex-specific differentiation during peri-pubertal life (e.g., Ahmed et al., 2008; Koshibu et al., 2004; Yildirim et al., 2008). Levels of oestrogen receptor (ER1), as well as androgen receptor (AR), were evaluated, as conversion of testosterone to oestrogen via aromatase during the pubertal period has been suggested to impact upon later social interactions (Kellogg and Lundin, 1999).

Gonadal hormones are known to interact extensively with the hypothalamic–pituitary–adrenal (HPA) axis during early life and adulthood (Handa and Weiser, 2014; Romeo, 2010), and recent studies have shown that developmental changes in HPA axis reactivity are sensitive to the organizational effects of gonadal hormone exposure during both the perinatal and peri-pubertal stages of life (e.g., Evuarherhe et al., 2009; Goel and Bale, 2008). For example, male rats that have been castrated after puberty exhibit testosterone-induced suppression of corticosteroid secretion when undergoing stress in adulthood, while pre-pubertally castrated males do not exhibit testosterone-induced suppression of HPA axis activation (Evuarherhe et al., 2009). Pubertal gonadal hormone exposure could potentially impact upon later behavioural and physiological responses to aversive environments and novel stimuli via modulation of corticotropin-releasing factor, CRF_2_, receptors (Panagiotakopoulos and Neigh, 2014), which are located in numerous areas of the brain, including the hippocampus, amygdala and various regions of the hypothalamus (Chalmers et al., 1995). CRF_2_ receptor density has recently been shown to increase during puberty in the medial amygdala of male, but not female, rats (Weathington and Cooke, 2012), and this sex difference could relate to circulating levels of gonadal hormones. Therefore, the effects of pre- and post-pubertal castration on mRNA expression of CRF-R2 were also examined in this study.

In summary, male rats were castrated either before puberty (postnatal day, *pnd*, 34/35) or after puberty (pnd 58/59) and were tested on four behavioural tasks, namely an OF, EPM, LD box and object/social novelty task, in adulthood (pnd 101–110). A pilot study was undertaken to establish baseline male responses to the LD box compared to females, given that previous studies in our laboratory have already provided baseline responses in the OF and EPM apparatus (Lynn and Brown, 2009, 2010). Expression of AR, ER1 and CRF-R2 mRNA was then quantified in the hypothalamus, hippocampus (CA1) and medial amygdala of pre- and post-pubertally castrated males, using quantitative real-time PCR (qPCR).

## Methods

### Study 1: sex differences in behaviour in a light–dark box

#### Subjects and housing

The subjects were 12 male and 12 female rats, bred in-house from stock animals (purchased from Harlan, U.K.). Pups were weaned into same-sex sibling groups on pnd 24, then housed as same-sex pairs from pnd 26 onward in plastic and wire-mesh cages (52 cm × 40 cm × 26 cm) with ad libitum access to soy-free pellets and water. Housing rooms were maintained on a 12-hour light:dark cycle (lights on 07:00) and were controlled for temperature (20 ± 1 °C) and humidity (55 ± 5%). All appropriate guidelines and regulations were adhered to, as set out in the Principles of Laboratory Animal Care (NIH, Publication No. 85–23, revised 1985) and the UK Home Office Animals (Scientific Procedures) Act 1986.

#### Behavioural testing and apparatus

All subjects were tested in a light–dark (LD) box on pnd 95 or 96, with the two sexes counterbalanced across days. The LD apparatus consisted of a rectangular arena constructed from transparent perspex and separated into two sections using a grey perspex divider with an aperture at floor level. The larger, ‘light’ section (70 cm × 46 cm × 44 cm) was covered externally with thick white paper and illuminated with white light from above, while the smaller, ‘dark’ section (48 cm × 46 cm × 44 cm) was covered externally with thick black paper and enclosed with a lid. The apparatus was placed in a testing room and surrounded by a black curtain, with a video camera overhead. At the start of a testing session, a subject was transported in an enclosed box to the testing room and placed into the dark section of the arena. Each test lasted 5 min, and the *latency to first emerge from the dark area*, *time spent in the light area* and *number of transitions between the dark and light sections* were recorded from the live video footage. After the test, the subject was returned to the home cage, and the apparatus was cleaned using 70% alcohol before the next test.

### Study 2: pre-pubertal and post-pubertal castration

#### Subjects, housing and surgical procedure

The subjects were 20 male Lister hooded rats, bred in-house from stock animals (purchased from Harlan, U.K.). Weaning schedules and housing conditions were the same as described above, and all experimental procedures were conducted under UK Home Office licences. One group of males (N = 10) was castrated prior to the onset of puberty on pnd 33/34, and one groups of males (N = 10) was castrated after puberty on pnd 58/59. Castrations were carried out under isoflurane anaesthesia via a mid-line scrotal incision that was closed using metal clips, and each subject was anesthetized for around 15–20 min in total. Pain relief was provided pre-operatively (Carprofen) and during recovery (Metacam); and the clips were removed from all subjects three weeks later under light anaesthesia.

#### Weighing, behavioural testing and apparatus

All subjects were weighed weekly from pnd 21 to pnd 77. Behavioural testing took place in the same testing room as described for Study 1, with the apparatus surrounded by a black curtain and monitored using a ceiling-mounted video camera. All behavioural tests lasted 5 min, and the apparatus was cleaned with 70% alcohol between tests. All subjects were tested in each apparatus at a specific age starting with the EPM, given that this task is particularly sensitive to prior testing experience (e.g., Fernandes and File, 1996).

i) The *elevated plus*-*maze* (EPM) (pnd 101) was constructed from grey-painted wood and consisted of four arms (51 cm × 11 cm), arranged in a ‘plus’ shape and raised above the ground (56 cm) on a metal base. Two of the arms had enclosing walls (40 cm high; ‘closed arms’), and the other two arms had no walls (‘open arms’), and the arms were connected by a square, central area. The subject was placed into a closed arm at the beginning of the test, and the *time spent in the open arms*, *time spent in the closed arms*, *number of open arm entries*, *number of closed arm entries*, *total number of arm entries*, and *frequency of head*-*dipping* were recorded from the live video footage.
ii) The *LD box* (pnd 103) was described above, and the same behavioural measures were recorded as in Study 1.
iii) The *open field* (OF) (pnd 108) consisted of an arena (122 cm × 122 cm × 50 cm) constructed from grey-painted wooden walls and a floor, with a marked-out central area (61 cm × 61 cm). The subject was placed beside a wall at the beginning of the test, and the *total distance travelled*, *time in the central area*, and *number of entries to the central area* were calculated using Ethovision (Noldus, The Netherlands) software.
iv) The *object*/*social novelty* (OSN) *task* (pnd 110) was conducted in a perspex arena (118 cm × 46 cm × 44 cm) that contained two tall, transparent perspex boxes (20 cm × 23 cm x 45 cm), located in opposite corners of the arena. The arena was visually divided into three areas (middle section: 22 cm × 46 cm × 44 cm; two end sections: 48 cm × 46 cm × 44 cm) from above. A novel object (one of five items that were approximately 10 cm tall and made of ceramic or plastic) was placed into one of the boxes, and an unfamiliar female conspecific was placed into the other box immediately prior to the test. The subject was placed into the central area at the beginning of the test and the *time spent investigating the box containing the novel object*/*social partner* (i.e., subject makes physical contact with the box, with a bout ending when the subject moves 3 cm away from the box) and *time spent in each of the three areas* was recorded from the recorded video footage.

#### qPCR

All subjects were culled on pnd 137/138 via terminal anaesthesia, and brains were extracted immediately, maintained on dry ice until frozen and stored at − 80 °C. A metallic brain matrix was used to cut two coronal 1 mm slices with razor blades in order to extract samples using micro-dissection techniques from the ventromedial hypothalamus, CA1 area of the hippocampus and the medial amygdala (based on Paxinos and Watson, 1998). For each region, 1 mm punches were extracted from each hemisphere and combined, then immediately stored at − 80 °C. Total mRNA from the tissue was extracted using Absolutely RNA Miniprep kits (Agilent Technologies, Santa Clara, CA, USA) according to the manufacturer's instructions. The quantity and integrity of RNA were assessed with an RNA 6000 Pico assay kit using the Agilent 2100 bioanalyzer (Agilent Technologies) according to the manufacturer's instructions. The mean RIN number for these samples was 8.3 ± 0.6 (SD). First strand cDNA was synthesized using Affinity Script Multiple Temperature cDNA Synthesis kits (Agilent Technologies) following the manufacturer instructions and diluted to obtain a final concentration of 30 pg·μl-1.

The obtained cDNA was used to perform qPCR for AR, ER1, CRF-R2 and the house-keeping gene, Cytochrome c1 (CYC1), for the three selected brain regions using gene-specific primers. CYC1 was determined as the best candidate house-keeping gene (M = 1.175, all other candidates M > 1.175) using a rat GeNorm kit (PrimerDesign, Southampton, UK). Specific SYBR Green primers were designed (by PrimerDesign) based on published rat nucleotide gene sequences — AR sense primer: CGTCCTCACTGTCTCTGTATAAG, anti-sense primer: GAGCGAGCGGAAAGTTGTAG (GenBank accession no. NM_012502); ER1 sense primer: GCCCTCCCGCCTTCTACAG, anti-sense primer: CATAGTCGTTACACACAGCACAGTA (GenBank accession no. NM_012689); CRF-R2 sense primer: GAAACTCAGAGCCCAAGTACG, anti-sense primer: CTTCCTCTTCTCCTTTCTCTTCTC (GenBank accession no. NM_031019). All qPCR reactions were run in duplicate and were performed in 20 μl reactions containing 10 μl of Precision FAST Master Mix (Agilent technologies), 1 μl of specific SYBR Green primer (PrimerDesign) at a working concentration of 300 nM, 4 μl of RNAse/DNAase-free water and 5 μl of appropriate cDNA along with no-template controls and blanks. Reactions were performed on a Stratagene MX 3005P (Agilent Technologies) at 95 °C for 1 min, then 50 cycles of 95 °C for 5 s and 60 °C for 20 s. From standard curves generated with known concentrations of cDNA, the amplification efficiency (Eff = 10(− 1/slope)− 1) was determined to be 87% for AR, 100% for ER1, and 96% for CRF-R2. The Delta Ct method (ΔCt) was used to quantify the expression of AR, ER1, and CRF-R2 relative to CYC1: 2-(Ct Gene − Ct CYC1).

### Statistical analyses

All analyses were conducted in SPSS (Version 22) and G*Power (Version 3.1). After testing that the data fit the assumptions of parametric statistics, body weight data were analysed using a repeated-measures ANOVA, followed by simple effects post-hoc tests, and behavioural data were analysed using multivariate ANOVAs. The qPCR data were analysed using repeated-measures ANOVAs with the Greenhouse Geisser correction, as sphericity was violated, followed by least significant difference post-hocs with a Bonferroni correction for multiple comparisons. Effect sizes were calculated as partial eta squared (*η*_*p*_^2^) for main effects and interactions in ANOVAs, or as Cohen's *d* for pair-wise comparisons. All data are presented as means and SEMs.

## Results

### Study 1

**Fig. 1:**
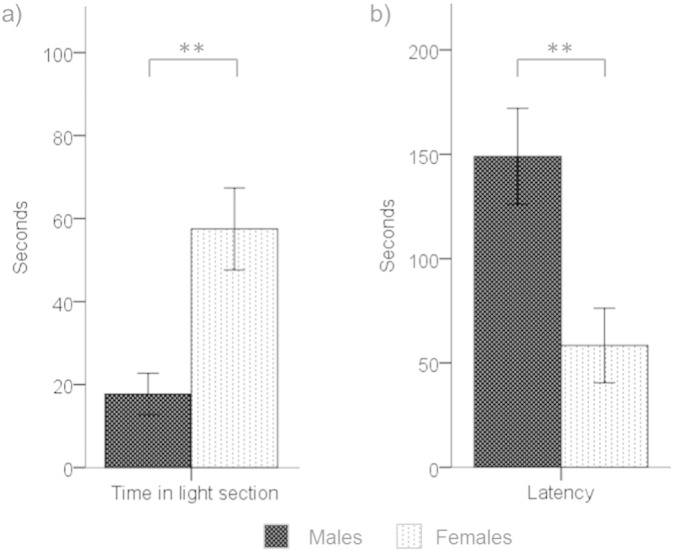
a) Time spent in the light section of LD box and b) latency to enter light section by males (black bars) and females (stippled bars) (means and SEMs; ** = p < 0.01).

Males spent less time in the light section of the LD box (*F*_1,22_ = 12.95, p < 0.01,*η*_*p*_^2^ = 0.37), and exhibited a longer mean latency to enter the light section (*F*_1,22_ = 9.70, p < 0.01,*η*_*p*_^2^ = 0.31), than females (Fig. 1).

### Study 2

#### Body weight

There was a significant interaction between age and treatment group on adult body weight (age: *F*_8,144_ = 2917.27, p < 0.01,*η*_*p*_^2^ = 0.99; treatment group: *F*_1,18_ = 1.69, p = 0.21,*η*_*p*_^2^ = 0.09; age x treatment group: *F*_8,144_ = 3.46, p = 0.001,*η*_*p*_^2^ = 0.16). Post-hoc simple effects tests revealed that pre-pubertally castrated males had a lower average body weight than post-pubertally castrated males on pnd 56 only (p < 0.05, *d* = 0.92).

#### EPM

**Fig. 2:**
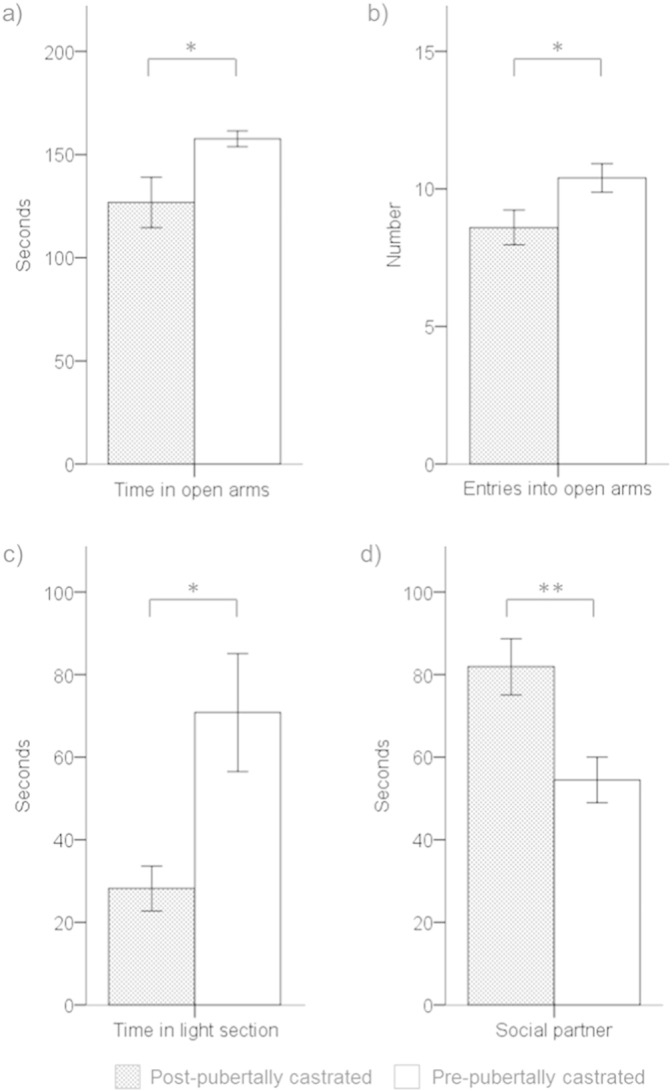
a) Time spent on the open arms of the EPM (seconds), b) number of entries into the open arms of the EPM (number of times/test), c) time spent in the light section of LD box (seconds), and d) time spent investigating a chamber that contained a novel opposite-sex social partner (seconds) by post-pubertally (grey bars) and pre-pubertally (white bars) castrated males (means and SEMs; * = p < 0.05, ** = p < 0.01).

Males that had been castrated before puberty spent more time on the open arms of the EPM (*F*_1,18_ = 5.86, p = 0.03,*η*_*p*_^2^ = 0.25; Fig. 2a), and entered the open arms more frequently (*F*_1,18_ = 4.80, p = 0.04,*η*_*p*_^2^ = 0.21; Fig. 2b), than males that had been castrated after puberty. The frequency of head-dipping was also higher for males castrated pre-pubertally (12.3 ± 0.7 number of times/test) than males castrated post-pubertally (8.2 ± 0.9) (*F*_1,18_ = 12.85, p < 0.01,*η*_*p*_^2^ = 0.42). The two groups of males did not differ in time spent in the closed arms (*F*_1,18_ = 3.15, p = 0.09,*η*_*p*_^2^ = 0.15; post-pubertally castrated: 84.6 ± 9.6 s; pre-pubertally castrated: 65.0 ± 5.5 s), number of closed arm entries (*F*_1,18_ = 0.48, p = 0.50,*η*_*p*_^2^ = 0.03; post-pubertally castrated: 7.4 ± 0.6 times per test; pre-pubertally castrated: 6.9 ± 0.4), or total number of arm entries (*F*_1,18_ = 1.73, p = 0.20,*η*_*p*_^2^ = 0.09; post-pubertally castrated: 16.0 ± 0.70 times per test; pre-pubertally castrated: 17.3 ± 0.70 times per test).

#### LD box

Males that had been castrated before puberty spent more time in the light area of the LD box than males that had been castrated after puberty (*F*_1,18_ = 7.74, p = 0.01,*η*_*p*_^2^ = 0.30; Fig. 2c), while the latency to enter the light area did not differ significantly between treatment groups (*F*_1,18_ = 1.37, p = 0.26,*η*_*p*_^2^ = 0.07; post-pubertally castrated: 70.8 ± 29.9 s; pre-pubertally castrated: 33.4 ± 11.4 s).

#### OF

The total distance travelled in the OF did not differ significantly between the treatment groups (*F*_1,18_ = 0.62, p = 0.44,*η*_*p*_^2^ = 0.03; post-pubertally castrated: 3.9 ± 0.2 m; pre-pubertally castrated: 3.7 ± 0.2 m), nor did time spent in the central area (*F*_1,18_ = 0.22, p = 0.64,*η*_*p*_^2^ = 0.01; post-pubertally castrated: 20.7 ± 2.4 s; pre-pubertally castrated: 25.6 ± 10.0 s) or latency to enter the central area (*F*_1,18_ = 0.98, p = 0.34,*η*_*p*_^2^ = 0.05; post-pubertally castrated: 22.0 ± 4.8 s; pre-pubertally castrated: 14.9 ± 5.4 s).

#### OSN task

Males that had been castrated before puberty spent less time investigating the chamber containing the female conspecific than males that had been castrated after puberty (*F*_1,18_ = 9.77, p < 0.01,*η*_*p*_^2^ = 0.35; Fig. 2d). No significant difference was found between treatment group for time spent investigating the novel object (*F*_1,18_ < 0.01, p = 0.98,*η*_*p*_^2^ < 0.01; post-pubertally castrated: 29.1 ± 4.6 s; pre-pubertally castrated: 29.3 ± 4.6 s). Neither time spent in the section of the arena containing the social partner (*F*_1,18_ = 2.09, p = 0.17,*η*_*p*_^2^ = 0.10; post-pubertally castrated: 148.5 ± 8.0 s; pre-pubertally castrated: 127.9 ± 11.9 s), nor time spent in the section of the arena containing the novel object (*F*_1,18_ = 1.36, p = 0.26,*η*_*p*_^2^ = 0.07; post-pubertally castrated: 95.2 ± 7.0 s; pre-pubertally castrated: 108.9 ± 9.5 s), differed between treatment group.

#### qPCR

Although AR levels appeared to be higher in post-pubertally, than in pre-pubertally, castrated males, AR levels did not differ significantly between treatment groups (*F*_1,18_ = 1.77, p = 0.20,*η*_*p*_^2^ = 0.09), and the interaction between treatment group and brain area was also non-significant (*F*_1.4,26.0_ = 0.13, p = 0.81,*η*_*p*_^2^ = 0.01; Table 1). Similarly, levels of ER1 and CRF-R2 did not differ between treatment groups (ER1: *F*_1,18_ = 0.06, p = 0.80, *η*_*p*_^2^ < 0.01; CRF-R2: *F*_1,18_ = 1.55, p = 0.23, *η*_*p*_^2^ = 0.08), and the interactions between treatment group and brain area were also non-significant for these two receptors (ER1: *F*_1.4,24.3_ = 1.81, p = 0.19, *η*_*p*_^2^ = 0.09; CRF-R2: *F*_1.2,21.0_ = 0.03, p = 0.09, *η*_*p*_^2^ < 0.01). The main effect of brain area was non-significant for both AR (*F*_1.4,26.0_ = 0.66, p = 0.48,*η*_*p*_^2^ = 0.04) and ER1 (*F*_1.4,24.3_ = 1.44, p = 0.25,*η*_*p*_^2^ = 0.07). In constrast, differences in CRF-R2 levels between brain areas were marginally significant (*F*_1.2,21.0_ = 3.99, p = 0.05, *η*_*p*_^2^ = 0.18). While planned post-hoc comparisons showed that CRF-R2 levels were higher in the ventromedial hypothalamus than in the hippocampus (p = 0.04, *d* = 0.70), the difference was non-significant when a Bonferroni correction was applied (p = 0.13, *d* = 0.70), and all other pairwise comparisons were non-significant (ps > 0.05, *d*s ≤ 0.44)

## Discussion

The main finding was that male rats that had been castrated before puberty spent more time on the open arms of the EPM, and in the light section of the LD box, than males that had been castrated after puberty. A lack of exposure to testicular hormones during the peri-pubertal period therefore led to more female-typical behavioural responses to novel environments in adulthood, in terms of time spent in the more aversive areas of these two environments. Previous studies have consistently shown that female rodents spend more time on the open arms of EPMs (e.g., Johnston and File, 1991; Lynn and Brown, 2009, 2010), and our pilot study confirmed that female rats spend more time than males in the light area of the LD box, as previously reported (e.g., Hughes et al., 2004). In contrast, the measures of locomotor activity (i.e., distance travelled in OF, number of closed arm entries in EPM) did not differ between the treatment groups, which suggests that behavioural differences in response to the aversive areas cannot be attributed solely to treatment effects on motor activity, supporting the null results of previous studies (e.g., Bengelloun et al., 1976; Stewart and Cygan, 1980). Males castrated after puberty spent more time investigating an unfamiliar female than pre-pubertally castrated males, in line with previous research showing that neural maturation during the peri-pubertal period is required for males to exhibit adult sexual responsiveness (e.g., Bell et al., 2013; Schulz et al., 2004). As hormone receptor levels did not differ significantly between the treatment groups in the brain regions examined, the neuroendocrine mechanisms underlying the long-term effects of pubertal testicular hormone exposure on response to novel environments have yet to be fully elucidated.

Time spent in the exposed or brightly lit areas of novel environments is considered to reflect an animal's underlying negative emotional state, and animals that spend more time in the bright or exposed areas are commonly described as showing a lower anxiety-like response than other subjects (e.g., Bourin et al., 2007; Walf and Frye, 2007), based on the assumption that locomoting in exposed spaces increases the risk of predation in the rodent's natural habitat (Nestler and Hyman, 2010). Therefore, removal of testicular hormones during the peri-pubertal period appears to reduce the anxiety-like responses of males in later life. Consistent with these findings, peri-pubertal administration of testosterone to intact male hamsters has been shown to reduce time spent on the open arms of an EPM in adulthood (Morris et al., 2013). The elevated levels of social interaction shown by pre-pubertally castrated male rats when tested in a novel environment (Primus and Kellogg, 1989, 1990) could also potentially reflect a reduced anxiety-like response to novel environments in these males, if social interactions are more likely to occur when an animal is in a positive emotional state. Removal of testicular hormones during the first few days of life in rats has also been reported to increase time spent in the exposed areas of novel environments when tested in adulthood (e.g., Lucion et al., 1996; Zuloaga et al., 2011), while treatment of females with testosterone during this period has the opposite behavioural effects (Swanson, 1966). Thus, the organizational effects of testicular hormone exposure during the peri-natal and peri-pubertal periods are similar and are potentially mediated by testosterone following conversion to oestrogen via aromatase (Kellogg and Lundin, 1999; Patisaul and Bateman, 2008; Zuloaga et al., 2011).

However, whether the time spent in exposed or brightly lit areas of novel environments should necessarily be interpreted as reflecting an animal's anxious state can be questioned, as other factors, such as motivation to escape, can also impact upon behavioural responses (Johnston and File, 1991). Female rodents, in addition to spending more time than males in the aversive areas of novel environments, exhibit more robust behavioural responses in conflict tasks, such as punished drinking and acoustic startle response tests, and more defensive behaviour when exposed to predator cues than males (Blanchard et al., 1991; Kokras and Dalla, 2014), which suggests that male and female rodents differ in coping strategies when exposed to stressors (e.g., Steenbergen et al., 1990). Therefore, the organizational effects of testicular hormones during the peri-pubertal period could alternatively be described as impacting upon adult coping strategies, such that normal exposure of males to testicular hormones results in novelty-induced behavioural suppression in adulthood. Female rodents also commonly exhibit greater activation of the HPA axis than males when exposed to a stressor (e.g., Handa et al., 1994; Seale et al., 2004), and removal of testicular hormones during early neonatal or peri-pubertal periods enhances HPA axis reactivity in male rats (e.g., Evuarherhe et al., 2009; Morales et al., 2014; Zuloaga et al., 2011). Our results therefore provide evidence that, in addition to suppressing later physiological stress responses, exposure of males to testicular hormones during early sensitive periods leads to reactive, rather than proactive, behavioural strategies in novel environments.

Administration of testosterone to adult male rodents has been shown to increase time spent in the aversive areas of novel environments (e.g., Bitran et al., 1993; Seale et al., 2004), while castration in adulthood has the opposite effects (e.g., Frye and Seliga, 2001; Khakpai, 2014; Seale et al., 2004). The activational effects of testosterone on the behavioural response to novel environments in adulthood therefore appear to be opposite to the organizational effects during early life. The mechanisms responsible for these differential effects of testosterone during early periods of life compared to adulthood are not known (McHenry et al., 2014). While gonadal hormone exposure can have long-term impacts on behaviour via modulation of steroid hormone receptors levels and via other neurodevelopmental processes (Juraska et al., 2013), the current study did not find any significant effect of peri-pubertal hormone manipulation on AR, ER1 or CRF-R2 levels in the hypothalamus, hippocampus or amygdala. These findings are consistent with a previous study on hamsters that reported no differential effect of pre-pubertal versus post-pubertal castration on AR and ER immunoreactivity in the medial amygdala and ventromedial hypothalamus (Romeo et al., 2000). This previous study also reported that, when treated with testosterone in adulthood, AR levels were higher in the medial preoptic nucleus of pre-pubertally castrated, than post-pubertally castrated, males (Romeo et al., 2000), leaving open the possibility that some changes in hormone receptor levels are only revealed following administration of testosterone in later life.

In the current study, males that were castrated after puberty spent more time investigating the box containing a novel female conspecific than males that were castrated before puberty, suggesting that peri-pubertal exposure to testosterone organizes the neural mechanisms that underpin responses to visual, olfactory and auditory cues from female conspecifics. This result is consistent with previous studies on hamsters, which have reported that males castrated before puberty exhibit deficits in sexual behaviour when treated with testosterone in adulthood, in terms of low levels of mounts, intromissions and ejaculations, compared to post-pubertally castrated males (e.g., Schulz et al., 2004; Schulz and Sisk, 2006). Our results extend these previous studies by revealing that the effects of pre-pubertal castration on response to female partners were exhibited even in the absence of testosterone administration in adulthood. Pre-pubertally castrated hamsters have been shown to process vaginal chemosensory cues from females appropriately (De Lorme et al., 2012b), which suggests that low levels of sexual activity in pre-pubertally castrated males result from reduced sexual motivation, rather than a reduced ability to process sexual cues. In support of this hypothesis, testosterone administration to pre-adolescent male hamsters does not result in adult-like patterns of activation in the mesocorticolimbic dopamine system (Bell et al., 2013), which suggests that the rewarding properties of female sexual cues require neural maturation during the pubertal period. The results of the current study are consistent with the hypothesis that pre-pubertally castrated males experience a reduced reward from interacting with females compared to post-pubertally castrated males.

The two groups of subjects received the surgical procedure at different ages, which could have potentially impacted upon behavioural development independently from any organizational effects of testicular hormones. Exposure to general anaesthesia during early life has been shown to induce neurotoxicity in rodents and primates (Sanders et al., 2013), and the location or extent of neurotoxicity could potentially vary according the age of the animal. However, in the current study, exposure to isoflurane was relatively brief (15–20 min) compared to studies that have reported a link between anaesthesia and neurotoxicity (e.g., 6 h: Zhao et al., 2010), and a recent study showed that exposure of pre-pubertal rats to isoflurane for a short period (30 min) had no measurable effects on neuroapoptosis, dendritic spine density or dendritic length in the prefrontal cortex (Briner et al., 2010). While effects of anaesthesia on other brain areas cannot be ruled out, the lack of any significant differences in hormone receptor levels between the groups suggests that broad-scale neurodevelopmental trajectories were not altered in one group relative to the other, and the higher levels of CRF-R2 in the ventromedial hypothalamus than in the hippocampus are consistent with previous findings (Van Pett et al., 2000). The experimental design was directly comparable to that used in previous studies (e.g., De Lorme et al., 2012b; De Lorme and Sisk, 2013; Schulz et al., 2004), and no effects of pre-pubertal sham castrations on behaviour or brain function have yet been found (Morales et al., 2014; Schulz et al., 2006). As the time period between surgery and behavioural testing also differed between the groups, future studies will be required to confirm that the behavioural effects are maintained over time.

In summary, the results of this study are consistent with the hypothesis that the organizational effects of testicular hormone exposure during the peri-pubertal period extend beyond sexual behaviour and include response to novel environments. Exposure to testicular hormones during the peri-pubertal and early neonatal periods of life appear to induce a male-typical response to novel environments, in terms of reduced time spent in exposed areas and dampened HPA axis activity, which contrasts with the effects of testosterone on behavioural and neuroendocrine response to novel environments in adulthood. Future studies could potentially shed light on why early organizational effects of testicular hormones differ from activational effects on these types of behavioural tasks (McHenry et al., 2014). Gaining a greater understanding of the mechanisms involved in the organizational effects of testicular hormone exposure on response to novel environments and on functioning of the HPA axis will potentially have implications for understanding sex differences in susceptibility to mood disorders in human beings. Adolescence is a time of increased prevalence of these disorders (McLean and Anderson, 2009), and circulating gonadal hormones have been hypothesised to influence the likelihood of developing mood disorders during this key stage of life (Hyde et al., 2008; Naninck et al., 2011). While ovarian hormones have received the most research attention, testicular hormones potentially have long-term neuroprotective benefits for mood disorders (McHenry et al., 2014).

## Figures and Tables

**Table 1:** Relative levels of AR, ER1 and CRF-R2 gene expression in the VMH, CA1 of the hippocampus, and MA of post-pubertally and pre-pubertally castrated males (means and SEMs). * = p < 0.05 in post-hoc pair-wise comparison (compared to CA1).

	AR			ER1			CRF-R2		
	VMH	CA1	MA	VMH	CA1	MA	VMH	CA1	MA
Post-pubertally castrated	0.42 (0.15)	0.37 (0.11)	0.36 (0.15)	0.05 (0.01)	0.03 (0.02)	0.10 (0.07)	0.16 (0.06)	0.06 (0.03)	0.10 (0.04)
Pre-pubertally castrated	0.24 (0.07)	0.23 (0.09)	0.15 (0.06)	0.12 (0.07)	0.01 (0.00)	0.29 (0.01)	0.11 (0.05)	0.02 (0.01)	0.04 (0.02)
All subjects combined	0.33 (0.08)	0.30 (0.07)	0.25 (0.08)	0.08 (0.04)	0.02 (0.01)	0.07 (0.04)	0.13* (0.04)	0.04 (0.02)	0.07 (0.02)
